# Reduced endogenous secretory RAGE in blood and bronchoalveolar lavage fluid is associated with poor prognosis in idiopathic pulmonary fibrosis

**DOI:** 10.1186/s12931-020-01410-3

**Published:** 2020-06-11

**Authors:** Kakuhiro Yamaguchi, Hiroshi Iwamoto, Witold Mazur, Shinichiro Miura, Shinjiro Sakamoto, Yasushi Horimasu, Takeshi Masuda, Shintaro Miyamoto, Taku Nakashima, Shinichiro Ohshimo, Kazunori Fujitaka, Hironobu Hamada, Noboru Hattori

**Affiliations:** 1 Department of Molecular and Internal Medicine, Graduate School of Biomedical and Health Sciences, Hiroshima University, 1-2-3 Kasumi, Minami-ku, Hiroshima, 734-8551 Japan; 2 Heart and Lung Centre, Division of Pulmonary Medicine, University of Helsinki and Helsinki University Central Hospital, Helsinki, Finland; 3 Department of Emergency and Critical Care Medicine, Graduate School of Biomedical and Health Sciences, Hiroshima University, Hiroshima, Japan; 4 Department of Physical Analysis and Therapeutic Sciences, Graduate School of Biomedical and Health Sciences, Hiroshima University, Hiroshima, Japan

**Keywords:** esRAGE, IPF, BALF, Serum, Biomarker

## Abstract

**Background:**

The endogenous secretory receptor for advanced glycation end products (esRAGE) is a soluble isoform produced by alternative splicing of the RAGE gene. The isoform has anti-inflammatory properties due to its inhibition of the RAGE/ligand interaction and is reduced in the lung tissue of patients with idiopathic pulmonary fibrosis (IPF). This study aimed to investigate the association of esRAGE serum and bronchoalveolar lavage fluid (BALF) levels with progression of IPF.

**Methods:**

This study included 79 IPF patients and 90 healthy controls. IPF and control serum esRAGE levels were compared, and the correlation between serum and BALF esRAGE levels was analyzed in 57 IPF patient samples. We also investigated the relationship of esRAGE serum and BALF levels with prognoses and lung function parameters in patients with IPF.

**Results:**

Serum esRAGE levels in IPF patients were significantly lower than those in healthy controls (162.0 ± 102.4 ng/ml and 200.7 ± 107.3 ng/ml, *p* = 0.009), although the baseline characteristics of age and smoking history were not matched. Serum levels of esRAGE were correlated with BALF esRAGE levels (r_s_ = 0.317). The BALF esRAGE levels were also correlated with diffusion capacity for carbon monoxide (r_s_ = 0.406). A Kaplan-Meier curve analysis and univariate/multivariate Cox hazard proportion analysis revealed that lower levels of esRAGE in blood and BALF were significantly associated with poorer prognoses in patients with IPF.

**Conclusions:**

Decreased esRAGE levels in BALF and blood were associated with poor prognoses in patients with IPF. These results suggest that esRAGE could be related to the pathophysiology of IPF and serum esRAGE could be a potential prognostic marker of IPF.

## Background

The receptor for advanced glycation end-product (RAGE) is a transmembrane receptor that can bind numerous ligands, and the RAGE/ligand interaction results in cellular activation and gene transcription [1, 2]. Although the expression of RAGE is at low levels in the majority of normal tissues, it is abundantly expressed in the normal lung, especially in type 1 pneumocytes [3–5]. Idiopathic pulmonary fibrosis (IPF) is a progressive, fibrotic lung disease with poor prognosis, characterized by recurrent alveolar epithelial injury followed by aberrant tissue repair and excessive matrix deposition [6, 7]. Previous studies have shown that RAGE expression in lung tissue is reduced in IPF when compared with controls, and functional polymorphism of the RAGE gene is associated with risk of IPF [8–11]. Additionally, in RAGE knockout mice, pulmonary fibrosis is caused by aging and may be further aggravated by exposure to asbestos [12]; therefore, RAGE may play a homeostatic role in the lungs. These observations indicate that decreased expression of RAGE in the lungs is associated with the development and severity of IPF.

Alternative splicing of RAGE gene leads to the formation of endogenous secretory RAGE (esRAGE), which lacks the transmembrane domain but has an extracellular ligand-binding domain; it has anti-inflammatory properties and acts as a decoy neutralizing RAGE-ligands [13–17]. Interestingly, proteomic analysis demonstrated that, compared to control subjects, the expression of esRAGE was reduced in the lung tissue of patients with IPF and not in chronic obstructive pulmonary disease (COPD) [9]. This suggests that esRAGE may be related to the pathophysiology of IPF. On the other hand, there have been no studies investigating the association between circulatory esRAGE and the disease severity and prognosis of IPF.

This study aimed to investigate whether esRAGE concentrations in blood and bronchoalveolar lavage fluid (BALF) are associated with the prognoses of IPF, and to explore esRAGE regulatory mechanisms by focusing on RAGE gene polymorphisms. First, we measured esRAGE levels in the serum and BALF and evaluated their correlation. Second, we analyzed the association of esRAGE levels in the serum and BALF with pulmonary function parameters and prognosis in patients with IPF. Additionally, the correlation between esRAGE levels and differential cell counts in the BALF was investigated, because inflammatory cells in the lung are reported to express RAGE [18, 19]. Finally, an exploratory analysis investigated the association of esRAGE concentrations with two RAGE gene polymorphisms: rs1800624 and rs1800625, which influenced the promoter activity of the RAGE gene [20].

## Methods

### Subjects

A total of 79 IPF patients and 90 healthy control subjects were included in the present study. The patients first visited the Department of Respiratory Medicine in Hiroshima University Hospital (Hiroshima, Japan) from 2003 to 2015, and the diagnosis of IPF was confirmed on retrospective review based on the ATS/ERS criteria [7]. As a result, sixty-six IPF patients were diagnosed with usual interstitial pneumonia (UIP) pattern on high-resolution computed tomography (HRCT), and 13 IPF patients were diagnosed with possible UIP pattern on HRCT and histological UIP pattern in surgical lung biopsies. Additionally, BALF samples were obtained during bronchoscopy from 57 of the 79 patients with IPF. This study was approved by the Ethics Committee of Hiroshima University Hospital (IRB33 approved in 2001 and M326 approved in 2004), and all participants provided written informed consent.

### Serum and BALF measurements and pulmonary function tests

Serum and BALF samples were collected at diagnosis and stored at − 80 °C. BALF was obtained under local anesthesia by introducing 50 mL of saline into the lung and promptly drawing it out again via a bronchoscope [21]. These procedures were repeated three times, and, in total, 150 mL of saline was introduced. The target region (determined by the HRCT results) was either the middle lobe or the lingular segment, depending on which was most severely affected. All of the retrieved saline was mixed and centrifuged, and the supernatant was collected. esRAGE levels were measured with a commercially available enzyme-linked immunosorbent assay (ELISA) kits, according to the manufacturer’s instructions (esRAGE ELISA kit, B-Bridge International, Sunnyvale, CA, USA). The esRAGE levels in the serum and BALF were fully within the measurable range; this was confirmed by duplicate analysis.

Pulmonary function variables were measured using spirometry (Chestac-55 V and Chestac-8800, CHEST, Tokyo Japan) in accordance with the American Thoracic Society and the European Respiratory Society recommendations [22].

### DNA preparation and genotyping

Peripheral whole venous blood samples were gathered from all participants and stored at − 80°C. Genomic DNA was extracted with the phenol-chloroform extraction and ethanol precipitation methods, as previously described [23, 24]. The RAGE gene polymorphisms were genotyped successfully for all patients and healthy controls. The genotypes were determined using a commercially available polymerase chain reaction (PCR) assay. Each sample contained 10 μl of the PCR mixture, which in turn contained 0.25 μl of TaqMan SNP Genotyping Assay (C_3293837_1, C_8848033_1, C_2412456_10), 1.0 μl of genomic DNA (2.0 g/mL), 3.75 μl of double distilled water, and 5.0 μl of TaqMan Fast Universal PCR Master Mix. All aforementioned PCR mixture and genotyping assay reagents were from Life Technologies Corporation (Carlsbad, CA, USA). The real-time PCR analysis was conducted using the Applied Biosystems 7500 Fast Real-Time PCR System (Life Technologies Corp.).

### Statistical analyses

Values were expressed as mean ± standard deviation (SD). Differences between groups were identified using the Mann-Whitney U tests and Pearson’s chi-squared tests. The Spearman’s rank correlation coefficient was calculated to reveal any association of esRAGE BALF levels with esRAGE serum levels, lung function parameters, and differential cell counts in the BALF in patients with IPF. We used receiver operating characteristic (ROC) curve analysis to find the optimal value of serum and BALF esRAGE for predicting 5-year survival in patients with IPF. Survival was calculated with the Kaplan-Meier approach and the log-rank test. The Cox proportional hazards analysis was used to identify significant predictors of IPF prognosis. All *p*-values < 0.05 were considered significant. All data analyses were performed using JMP statistical software version 14.1.0 (SAS Institute Inc., Cary, NC, USA).

## Results

### Subject characteristics

Total study population baseline characteristics are shown in Table 1. Patients with IPF in both the serum and BALF analysis groups were significantly older, had higher pack-year smoking histories, and lower forced vital capacity (FVC) than the healthy controls. There were no significant differences in baseline characteristics between the IPF patient serum and BALF groups. Diffusion capacity for carbon monoxide (DLco) was measured in 73 of 79 IPF patients with serum sample and 55 of 57 patients with BALF sample, respectively; this was not correlated with age and smoking history in these patients (data not shown).

**Table 1:** Subject baseline characteristics

	Controls	Patients with IPF	
		Serum	BALF
Subjects, n	90	79	57
Age, years	53.8 ± 3.6	67.4 ± 8.8*	66.5 ± 8.8*
Sex, Male/Female	77/13	70/9	53/4
Smoking history, pack-years	16.8 ± 18.3	39.4 ± 28.5*	38.3 ± 25.0*
FVC, % predicted	109.3 ± 16.2	78.7 ± 18.2*	78.7 ± 19.2*
DLco, % predicted^a^	–	50.2 ± 16.3	51.2 ± 16.1
BALF total cell, × 10^4^/ml	–	–	21.9 ± 16.0
BALF macrophage, %	–	–	83.4 ± 11.4
BALF lymphocyte, %	–	–	11.4 ± 10.3
BALF neutrophil, %	–	–	3.3 ± 3.3
BALF eosinophil, %	–	–	1.9 ± 2.6

*vs. Control, *p* < 0.05 Mann-Whitney U-test

^a^DLco was measured in 73 of 79 IPF patients with serum sample and 55 of 57 patients with BALF sample, respectively

Values are mean ± SD unless stated otherwise

*BALF* bronchoalveolar lavage fluid, *DLco* diffusing capacity for carbon monoxide, *IPF* idiopathic pulmonary fibrosis, *FVC* forced vital capacity

### Baseline concentrations of esRAGE in blood and BALF

**Fig. 1:**
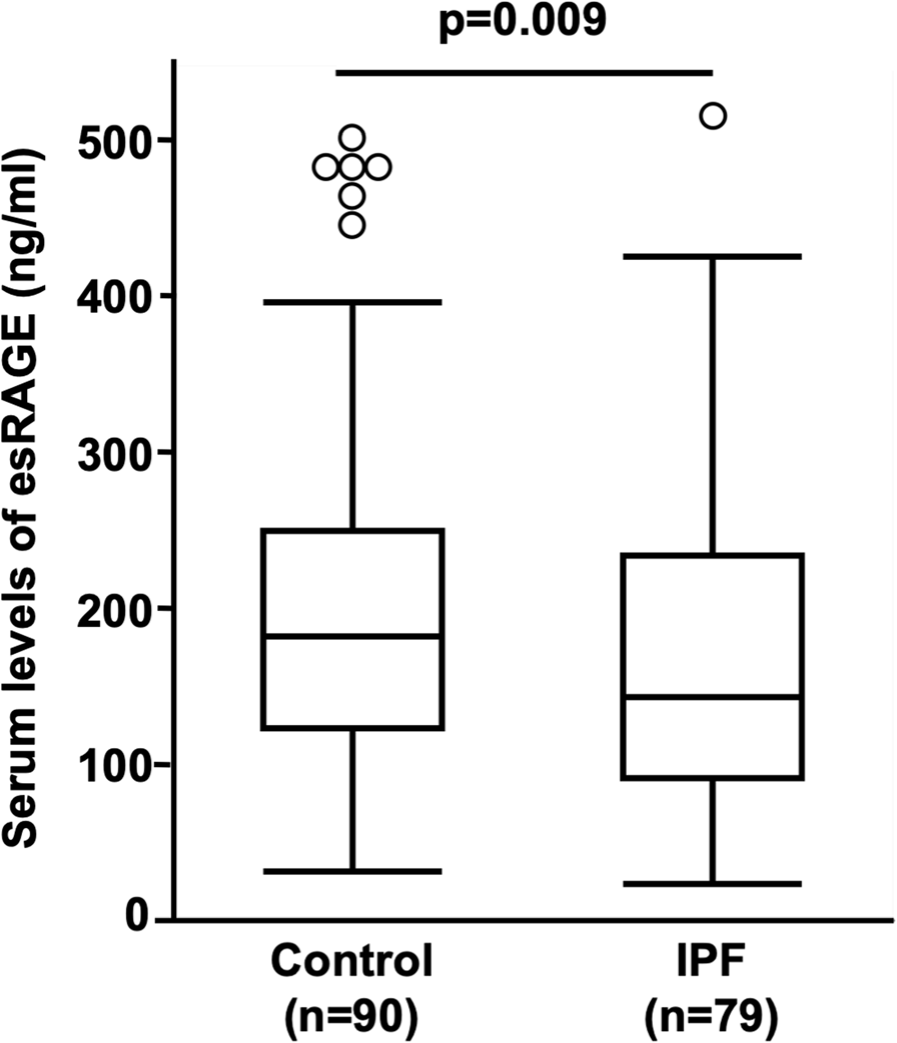
Serum levels of esRAGE in patients with IPF and control subjects The serum levels of endogenous secretory receptor for advanced glycation end products (esRAGE) in patients with idiopathic pulmonary fibrosis (IPF) were significantly lower than those in controls (162.0 ± 102.4 ng/ml and 200.7 ± 107.3 ng/ml, *p* = 0.009, respectively). Key: boxes represent the 25th to 75th percentiles; solid lines within the boxes show the median values; whiskers are the 10th and 90th percentiles; and the small circles represent outliers ^*^p < 0.05 (Mann-Whitney *U*-test) SD, standard deviation

Serum levels of esRAGE in patients with IPF were significantly lower than those in healthy controls (162.0 ± 102.4 ng/ml and 200.7 ± 107.3 ng/ml, respectively, *p* = 0.009, Fig. 1). However, there was no correlation of esRAGE levels in the serum with lung function parameters (data not shown).

**Fig. 2:**
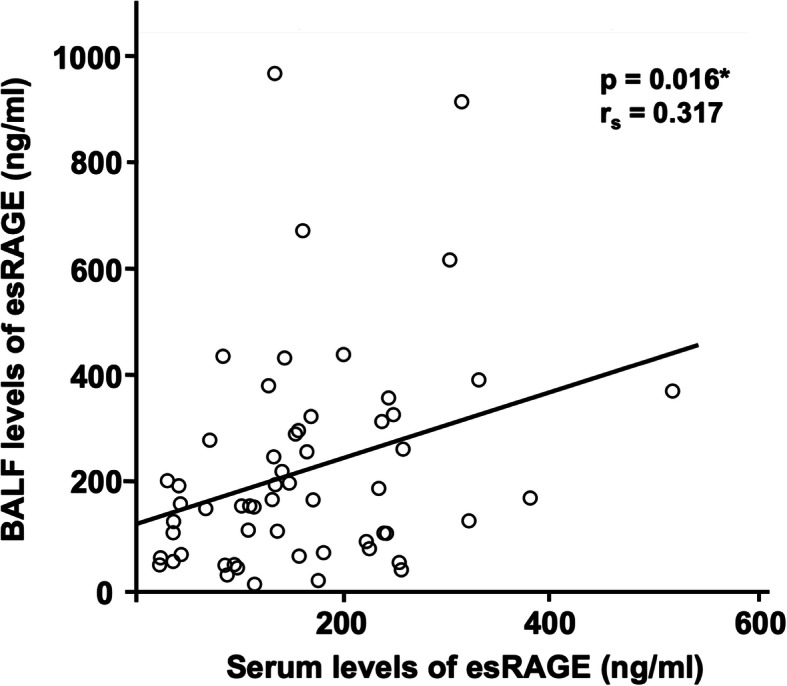
Association between the levels of esRAGE in serum and BALF The serum levels of endogenous secretory receptor for advanced glycation end products (esRAGE) were significantly correlated with bronchoalveolar lavage fluid (BALF) levels of esRAGE in patients with idiopathic pulmonary fibrosis (IPF). ^*^p < 0.05 (Spearman’s rank correlation coefficient)

The BALF levels of esRAGE were 219.7 ± 202.7 ng/ml, and positively associated with serum levels of esRAGE in patients with IPF (*r*_*s*_ = 0.317, *p* = 0.016; Fig. 2). The BALF levels of esRAGE were significantly correlated with DLco (*r*_*s*_ = 0.406, *p* = 0.002; Table 2), but not with FVC and differential cell counts in the BALF.

**Table 2:** Association of BALF esRAGE levels with pulmonary function parameters and differential cell counts in BALF (*n* = 57)

Variables	r_s_	*P*-value
Pulmonary function parameters		
FVC, % predicted	−0.025	0.853
DLco, % predicted^a^	0.406	0.002*
Differential cell counts in BALF		
Total cell, × 10^4^/ml	−0.002	0.990
Macrophage, %	0.060	0.667
Macrophage, × 10^4^/ml	0.016	0.907
Lymphocyte, %	0.125	0.369
Lymphocyte, × 10^4^/ml	0.160	0.247
Neutrophil, %	−0.049	0.727
Neutrophil, × 10^4^/ml	− 0.022	0.877
Eosinophil, %	−0.117	0.400
Eosinophil, × 10^4^/ml	−0.063	0.650

* *p* < 0.05 Spearman’s rank correlation coefficient

^a^DLco was measured in 55 of 57 patients with IPF

*esRAGE* endogenous secretory receptor for advanced glycation end products, *BALF* bronchoalveolar lavage fluid, *DLco* diffusing capacity for carbon monoxide, *FVC* forced vital capacity

In this study, the baseline characteristics of age and smoking history were not matched in patients with IPF and control subjects, and therefore we evaluated the association of esRAGE levels in the serum and BALF with these characteristics. There was no significant correlation of esRAGE levels with age and smoking history in patients with IPF (data not shown).

Furthermore, there was no significant difference in the blood and BALF esRAGE levels between patients with or without the minor allele of the RAGE gene polymorphisms (rs1800624 and rs1800625) (Additional file 1: Table S1).

### Prognostic value of esRAGE in blood and BALF

**Fig. 3:**
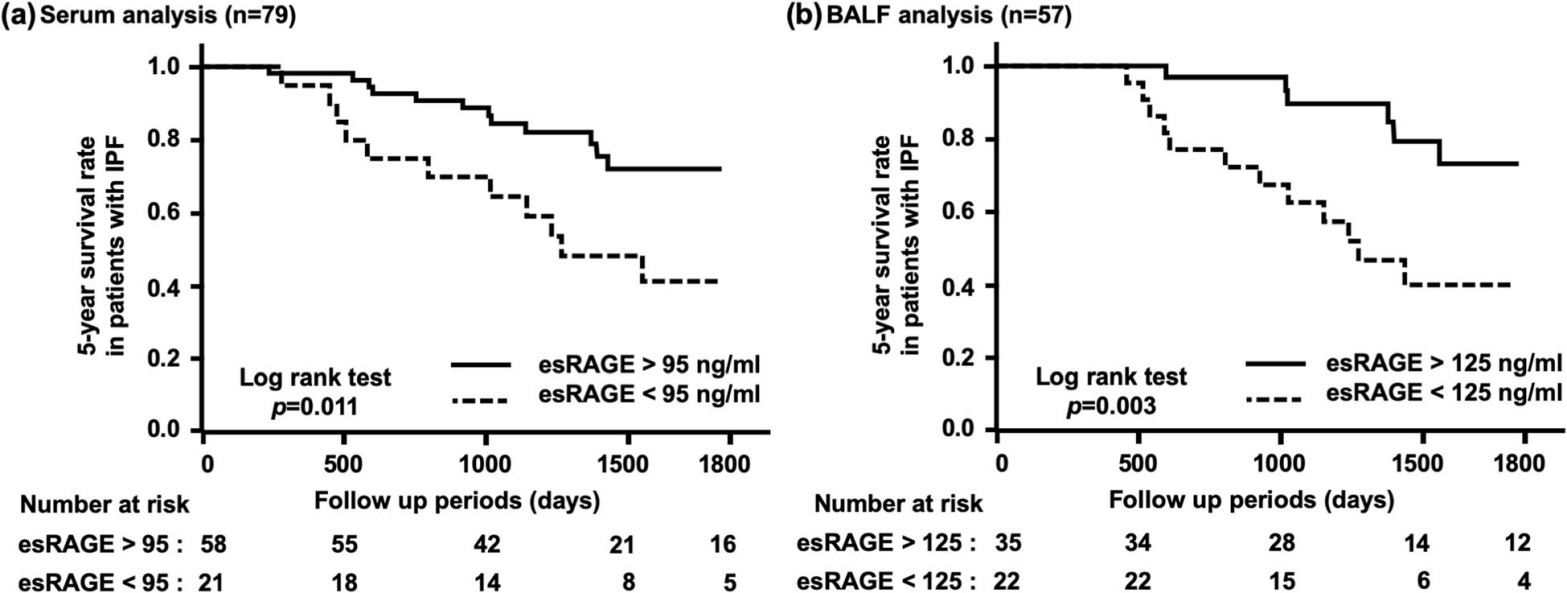
Five-year survival in patients with IPF based on the levels of esRAGE in serum and BALF. Decreased levels of endogenous secretory receptor for advanced glycation end products (esRAGE) both in (**a**) serum and (**b**) bronchoalveolar lavage fluid (BALF) were significantly associated with poorer survival in patients with idiopathic pulmonary fibrosis (IPF)

In patients with IPF, the median observation period was 42.2 months. The optimal cut-off levels for predicting 5-year survival rates were 95 ng/ml in the serum analysis group and 125 ng/ml in the BALF analysis group, as determined by the ROC curves with area under the curve (AUC) values of 0.716 and 0.717, respectively. The Kaplan-Meier method and log-rank test showed that the survival rates were significantly lower in patients with lower levels of esRAGE in both the serum and BALF analysis groups (*p* = 0.011 and *p* = 0.003, respectively; Fig. 3). Univariate and multivariate Cox proportional hazards analysis revealed that lower levels of esRAGE were significantly correlated with poorer prognoses in patients with IPF, in both the serum and BALF analysis groups (Table 3).

**Table 3:** Predicting value for 5-year mortality in IPF patients assessed by Cox proportional hazards model

Variables	Univariate analysis			Multivariate analysis		
	HR	95% CI	*p*-value	HR	95% CI	*p*-value
Serum (*n* = 79)						
Age, years	0.99	0.95–1.04	0.680	–	–	–
Sex, Male/Female	1.16	0.27–4.98	0.648	–	–	–
Smoking history, pack-years	1.00	0.98–1.01	0.825	–	–	–
FVC, % predicted	0.94	0.91–0.98	< 0.001*	0.94	0.91–0.98	0.002*
Serum esRAGE, ng/ml	0.99	0.98–0.99	0.019*	0.99	0.98–0.99	0.028*
BALF (n = 57)						
Age, years	1.02	0.97–1.08	0.474	–	–	–
Sex, Male/Female	0.94	0.12–7.09	0.953	–	–	–
Smoking history, pack-years	0.99	0.97–1.01	0.330	–	–	–
FVC, % predicted	0.96	0.93–0.99	0.009*	0.96	0.92–0.99	0.020*
BALF esRAGE, ng/ml	0.99	0.99–0.99	0.009*	0.99	0.98–0.99	0.041*

* *p* < 0.05 Cox proportional hazards model

*esRAGE* endogenous secretory receptor for advanced glycation end products, *BALF* bronchoalveolar lavage fluid, *CI* confidence interval, *IPF* idiopathic pulmonary fibrosis, *HR* hazard ratio, *FVC* forced vital capacity

## Discussion

This study demonstrated that serum esRAGE levels in IPF patients were significantly lower than those in healthy controls and that lower levels of serum esRAGE were associated with a poorer prognosis in IPF patients. Remarkably, decreased levels of BALF esRAGE were associated not only with decreased levels of serum esRAGE but also with lower DLco and poorer prognosis. These data could suggest that decreased serum levels of esRAGE may be associated with a pro-inflammatory condition of the lungs, which in turn decrease the levels of esRAGE in the BALF and result in a poor prognosis.

We found that lower levels of esRAGE in blood and BALF were independently associated with a poor prognosis in patients with IPF. RAGE-ligands such as HMGB1 and S100 proteins have been associated with the progression of IPF. In earlier reports, it has been shown that BALF and serum levels of HMGB1 are elevated in patients with IPF and that elevated serum HMGB1 levels are associated with acute deterioration and poor prognoses for IPF [25, 26]. Additionally, Xia et al. reported that the expression of the S100 protein A4 (S100A4) is increased in mesenchymal progenitor cells in patients with IPF, and increased S100A4 expression is associated with aberrant lung fibrosis in a bleomycin-induced mouse model [27]. Furthermore, esRAGE can act as a decoy receptor for RAGE ligands and inhibit the RAGE/ligand interaction, which may be associated with the IPF disease process [13–15, 28]. Therefore, the present results extend the previous proteomic study observations of decreased esRAGE expression in the lungs of patients with IPF [9], and indicate that decreased esRAGE may play a role in the progression of IPF.

The total pool of circulating soluble RAGE (sRAGE) consists of esRAGE and the ectodomain cell-surface RAGE released by metalloproteinases, such as matrix metalloproteinase 9 and a disintegrin and metalloprotease 10 [29]. In patients with COPD, sRAGE is down-regulated in lung tissue, while esRAGE is not, and reduced circulatory sRAGE is associated with the decline of lung function, while esRAGE is not [9, 30, 31]. However, this study showed that lower levels of circulatory sRAGE were associated with poor survival in patients with IPF, which was in line with the results of previous studies [8, 32], and we confirmed the association of the lung and circulatory esRAGE levels with decreased lung function and poor prognoses. These observations indicate that sRAGE and esRAGE might play different roles in the pathophysiology of lung diseases. However, the present results support the hypothesis that the RAGE/ligand interaction is related to the pathophysiology of IPF, but further investigation is warranted to elucidate causal relationships.

Serum levels of esRAGE were significantly reduced in patients with IPF compared to control subjects. Additionally, there was a significant positive correlation between serum and BALF esRAGE concentrations. These results are in line with those of the previous study elucidating decreased esRAGE expression in the lungs in patients with IPF [9], but its mechanism is still unknown. A possible explanation is that reduced esRAGE levels in IPF could result from lung parenchymal damage, because this study showed that decreased levels of esRAGE in BALF were related to reduced DLco, which was a sensitive indicator of the underlying parenchymal pathology [33–35]. Additionally, although inflammatory cells in the lungs are a potential source of esRAGE [18, 19], there was no correlation between esRAGE levels and differential cell counts in the BALF in this study. Another possible mechanism could be that increased RAGE/ligand interaction in the IPF lung may diminish esRAGE in the circulation [36]. Yet another possibility is that RAGE gene polymorphisms, which modulate the expression of esRAGE, could cause the altered levels of esRAGE in IPF. In order to test this hypothesis, we investigated the association of esRAGE concentrations with two RAGE gene polymorphisms that influence promoter activity (rs1800624 and rs1800625), but there was no significant correlation [20]. Further investigations are needed to elucidate the association between the esRAGE regulatory mechanism and lung fibrosis.

The present study had limitations. It was conducted in a single institution; therefore, the sample size was relatively small, especially with respect to procurement of BALF samples from IPF patients. Additionally, serum levels of esRAGE were significantly lower in patients with IPF than in healthy controls; however, the mean age of IPF patients was around 10 years more than that of healthy controls; further, more smokers were present among patients with IPF than controls. Although esRAGE levels in the serum and BALF were not significantly correlated with age and smoking history in this cohort (data not shown), this could have altered the results. Other studies to confirm the esRAGE circulatory prognostic cut-off level and its predictive value are necessary.

## Conclusions

In conclusion, decreased esRAGE levels in both BALF and blood were associated with a poor prognosis in patients with IPF. In particular, serum levels of esRAGE may be a potential prognostic marker reflecting dysregulated RAGE/ligand interactions in the lungs, indicated by lower BALF levels of esRAGE. Finally, further studies are warranted to investigate the role of the RAGE/ligand interaction effect on the progression of IPF.

## Supplementary information

**Additional file 1.**

## Data Availability

Please contact author for data requests.

## Abbreviations

BALF: Bronchoalveolar lavage fluid
CI: Confidence interval
COPD: Chronic obstructive pulmonary disease
DLco: Diffusion capacity for carbon monoxide
ELISA: Enzyme-linked immunosorbent assay
esRAGE: Endogenous secretory RAGE
HR: Hazard ratio
HRCT: High-resolution computed tomography
IPF: Idiopathic pulmonary fibrosis
PCR: Polymerase chain reaction
RAGE: Receptor for advanced glycation end products
ROC: Receiver operating characteristic
S100A4: S100 protein A4
SD: Standard deviation
sRAGE: Soluble RAGE
UIP: usual interstitial pneumonia
FVC: Forced vital capacity
