# Supplementary material for: Reduced endogenous secretory RAGE in blood and bronchoalveolar lavage fluid is associated with poor prognosis in idiopathic pulmonary fibrosis
Source: Respir Res. 2020 Jun 11;21:145. doi: 10.1186/s12931-020-01410-3 (PMC7291663; doi:10.1186/s12931-020-01410-3)
Supplement: Supplementary file 1 — Additional file 1. [file 12931_2020_1410_MOESM1_ESM.docx]

**Additional Table 1.**

|  |  |  |  | Control and IPF (n=167) | | | |  | Control (n=90) | | |  |
| --- | --- | --- | --- | --- | --- | --- | --- | --- | --- | --- | --- | --- |
|  |  |  |  | n | serum esRAGE | *p*-value |  | | n | serum esRAGE | *p*-value |  |
|  | rs184003 | C/C |  | 129 | 182.8 ± 105.3 |  |  | | 69 | 200.4 ± 106.1 |  |  |
|  |  | C/A & A/A |  | 38 | 183.7 ± 114.1 | 0.965 |  | | 21 | 201.4 ± 113.8 | 0.913 |  |
|  |  |  |  |  |  |  |  | |  |  |  |  |
|  | rs1800624 | A/A |  | 94 | 181.2 ± 110.2 |  |  | | 51 | 195.4 ± 110.2 |  |  |
|  |  | A/T & T/T |  | 73 | 185.4 ± 103.6 | 0.815 |  | | 39 | 207.6 ± 104.3 | 0.569 |  |
|  |  |  |  |  |  |  |  | |  |  |  |  |
|  | rs1800625 | A/A |  | 139 | 185.2 ± 107.0 |  |  | | 75 | 199.0 ± 108.4 |  |  |
|  |  | A/G & G/G |  | 28 | 172.4 ± 108.5 | 0.533 |  | | 15 | 208.9 ± 104.5 | 0.689 |  |

|  |  |  |  |  | IPF (serum n=77, BALF n=55) | | | | | |  |
| --- | --- | --- | --- | --- | --- | --- | --- | --- | --- | --- | --- |
|  |  |  |  | n | serum esRAGE | *p*-value |  | n | BALF esRAGE | *p*-value |  |
|  | rs184003 | C/C |  | 60 | 162.6 ± 101.6 |  |  | 44 | 173.3 ± 121.0 |  |  |
|  |  | C/A & A/A |  | 17 | 161.8 ± 114.1 | 0.941 |  | 11 | 296.3 ± 273.0 | 0.192 |  |
|  |  |  |  |  |  |  |  |  |  |  |  |
|  | rs1800624 | A/A |  | 43 | 164.3 ± 109.0 |  |  | 32 | 189.4 ± 156.2 |  |  |
|  |  | A/T & T/T |  | 34 | 160.0 ± 98.2 | 0.890 |  | 23 | 209.6 ± 184.2 | 0.621 |  |
|  |  |  |  |  |  |  |  |  |  |  |  |
|  | rs1800625 | A/A |  | 64 | 168.9 ± 103.8 |  |  | 43 | 203.2 ± 172.6 |  |  |
|  |  | A/G & G/G |  | 13 | 130.4 ± 100.9 | 0.183 |  | 12 | 178.8 ± 151.4 | 0.610 |  |

esRAGE, endogenous secretory receptor for advanced glycation end products; BALF, bronchoalveolar lavage fluid; IPF, idiopathic pulmonary fibrosis
